# Transoesophageal echocardiography as complementary imaging during pulsed field ablation of atrial fibrillation: a practical step-by-step approach

**DOI:** 10.1093/ehjimp/qyag155

**Published:** 2026-09-02

**Authors:** Federico Marchini, Matteo Arzenton, Federica Frascaro, Luca Canovi, Michele Malagù, Francesco Vitali, Sofia Meossi, Laura Rotondo, Gianluca Campo, Matteo Bertini, Rita Pavasini

**Affiliations:** Cardiology Unit, Azienda Ospedaliero Universitaria di Ferrara, Via Aldo Moro 8, Ferrara 44124, Italy; Cardiology Unit, Azienda Ospedaliero Universitaria di Ferrara, Via Aldo Moro 8, Ferrara 44124, Italy; Cardiology Unit, Azienda Ospedaliero Universitaria di Ferrara, Via Aldo Moro 8, Ferrara 44124, Italy; Cardiology Unit, Azienda Ospedaliero Universitaria di Ferrara, Via Aldo Moro 8, Ferrara 44124, Italy; Cardiology Unit, Azienda Ospedaliero Universitaria di Ferrara, Via Aldo Moro 8, Ferrara 44124, Italy; Cardiology Unit, Azienda Ospedaliero Universitaria di Ferrara, Via Aldo Moro 8, Ferrara 44124, Italy; Cardiology Unit, Azienda Ospedaliero Universitaria di Ferrara, Via Aldo Moro 8, Ferrara 44124, Italy; Cardiology Unit, Azienda Ospedaliero Universitaria di Ferrara, Via Aldo Moro 8, Ferrara 44124, Italy; Cardiology Unit, Azienda Ospedaliero Universitaria di Ferrara, Via Aldo Moro 8, Ferrara 44124, Italy; Cardiology Unit, Azienda Ospedaliero Universitaria di Ferrara, Via Aldo Moro 8, Ferrara 44124, Italy; Cardiology Unit, Azienda Ospedaliero Universitaria di Ferrara, Via Aldo Moro 8, Ferrara 44124, Italy

**Keywords:** atrial fibrillation, pulsed field ablation, transoesophageal echocardiography, pulmonary veins, transseptal puncture, procedural imaging

## Abstract

**Aims:**

Transoesophageal echocardiography (TOE) provides real-time soft-tissue anatomical information during pulsed field ablation (PFA) of atrial fibrillation. This focused technical report describes a standardized workflow in which TOE complements, rather than replaces, fluoroscopic, and angiographic guidance throughout the procedure.

**Technique:**

The workflow is organized into four consecutive phases: pre-procedural assessment, transseptal puncture, ablation–catheter configuration and positioning at the pulmonary vein ostia, and post-procedural assessment. Pre-procedural TOE focuses on exclusion of left atrial appendage thrombus, definition of pulmonary vein anatomy, assessment of the interatrial septum, and baseline evaluation for pericardial effusion. During transseptal access, orthogonal bicaval and short-axis views support puncture-site selection and continuous visualization of septal tenting, while fluoroscopy remains integral to guidewire and transseptal-system manipulation. During ablation, TOE complements fluoroscopic/angiographic guidance by depicting catheter configuration and its relationship with the pulmonary vein ostia and adjacent soft-tissue structures. At procedure completion, TOE is used to assess the iatrogenic interatrial septal defect and to exclude new pericardial effusion.

**Conclusion:**

TOE should be considered a complementary imaging modality during fluoroscopy- and angiography-guided PFA rather than a replacement for these techniques. Its principal contribution is real-time visualization of soft-tissue anatomy, transseptal puncture, catheter–tissue relationships, and immediate post-procedural complications. Prospective studies are required to quantify its incremental clinical value.

## Graphical Abstract

**Graphical Abstract:**
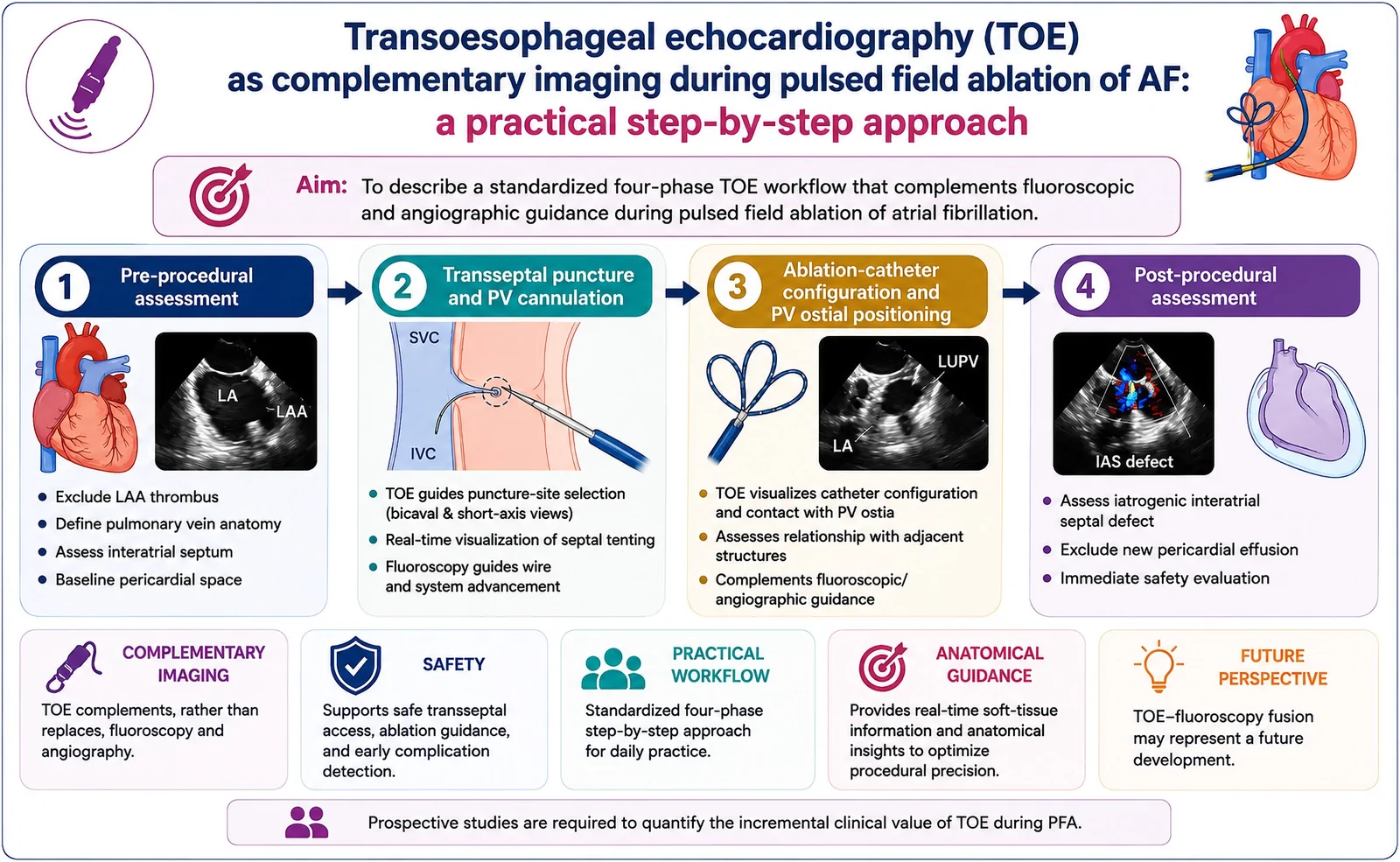

## Introduction

Pulsed field ablation (PFA) treats atrial fibrillation (AF) through irreversible electroporation. The pentaspline Farawave catheter (Farapulse™, Boston Scientific) delivers biphasic bipolar pulses in flower or basket configurations, accommodating different pulmonary vein (PV) anatomies.^1,2^

Transoesophageal echocardiography (TOE) can provide additional anatomical information during PFA, and initial experience supports the feasibility of its intraprocedural use, although comparative and prospective evidence remains limited.^3^ Intracardiac echocardiography (ICE) is an established intraprocedural imaging modality during AF ablation, providing real-time visualization of relevant anatomy and procedural guidance, whereas the role of TOE as a complementary imaging strategy during PFA has been less systematically defined.^4,5^ Building on the initial experience with TOE-guided PFA, the present report focuses on a granular and reproducible four-phase imaging workflow, linking specific TOE views and anatomical targets to each procedural step.

**Figure 1:**
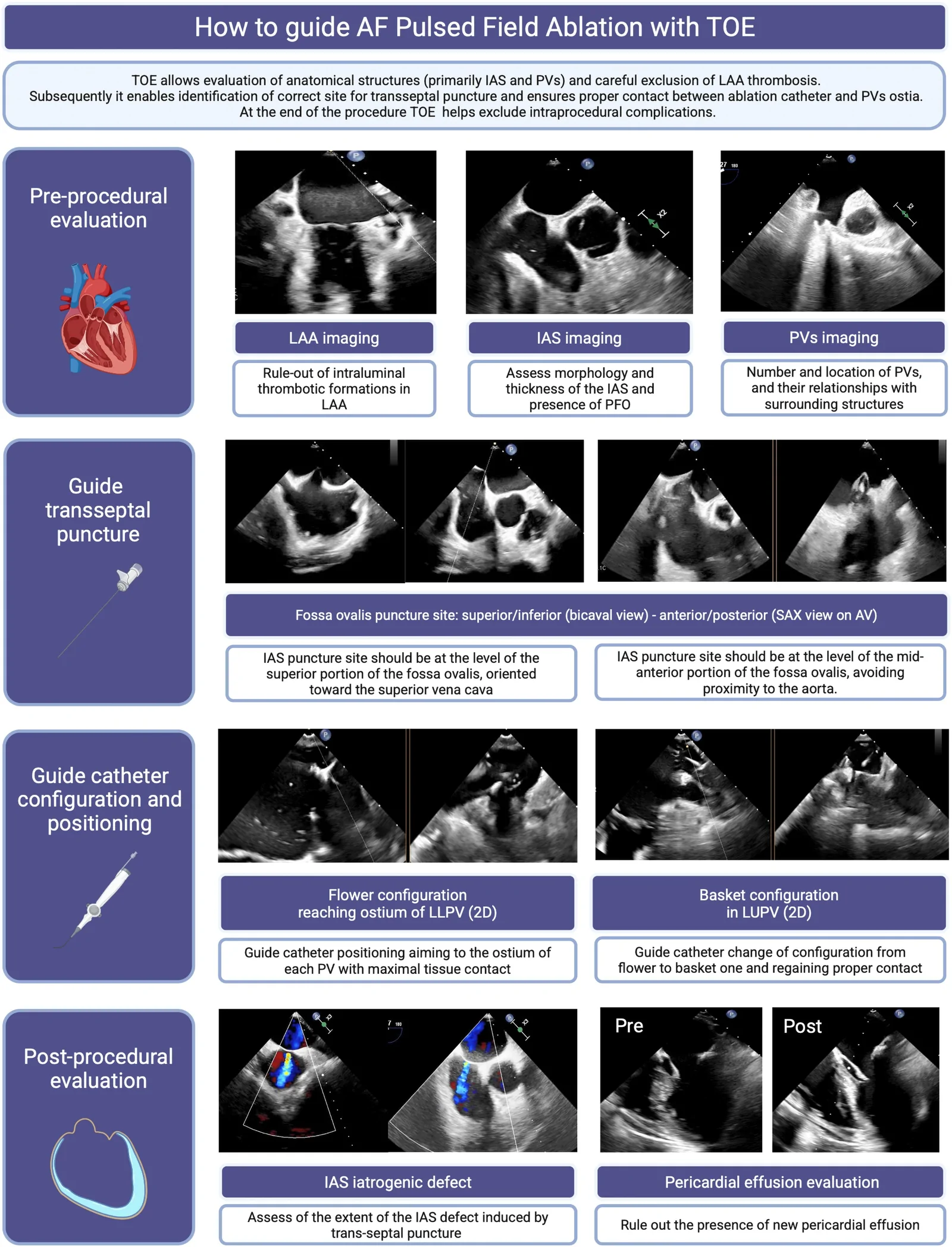
Step-by-step integration of TOE into fluoroscopy- and angiography-guided PFA. The figure illustrates the complementary role of TOE across the PFA workflow, including pre-procedural anatomical assessment, transseptal puncture, catheter configuration and positioning, and immediate post-procedural safety evaluation. TOE is used in conjunction with, rather than as a replacement for, fluoroscopic and angiographic guidance. SAX, short-axis view; AV, aortic valve; LLPV, left lower pulmonary vein; LUPV, left upper pulmonary vein.

TOE complements rather than replaces fluoroscopy or angiography by adding real-time soft-tissue information. This report presents a standardized workflow integrating TOE into conventional PFA guidance (*Figure 1*).

## Imaging protocol

### Step 1: pre-procedural assessment

A meticulous pre-procedural evaluation is essential to define baseline anatomy and optimize procedural safety. In the pre-procedural evaluation for PFA, it is necessary to assess the following:

- the presence of pericardial effusion before the procedure: baseline assessment of the pericardial space documents any pre-existing fluid and facilitates recognition of a new or increasing effusion during or after ablation, which may indicate a procedural complication.^5^
- Exclusion of intra-atrial thrombus: left atrial thrombus is a contraindication to AF ablation. Contemporary guidelines recommend avoiding interruption of oral anticoagulation in patients already anticoagulated before ablation; nevertheless, pre-procedural imaging to exclude thrombus remains relevant according to the clinical setting and local workflow.^6^ When TOE is performed, the left atrial appendage (LAA) should be assessed in multiple views—typically at 0°, 45°, 60°, 90°, and 135°—to visualize its lobes and recesses.^7^ LAA Doppler velocities, spontaneous echo contrast or sludge, and pericardial recesses around the appendage should also be considered.
- Pulmonary vein anatomy: evaluation of PV number, ostial location, and spatial orientation is relevant because PFA is primarily directed at PV isolation. TOE imaging is typically performed from the mid-oesophageal level. The left upper PV is commonly visualized at 45–90°, the left lower PV at 0° or 135°, and the right-sided PVs at 30–45° or 90–110°.^7^ Biplane imaging is particularly useful for defining ostial orientation and recognizing anatomical variants before ablation.
- Interatrial septal morphology: the interatrial septum (IAS) should be assessed for variants that may influence transseptal access, including atrial septal aneurysm, patent foramen ovale, and lipomatous hypertrophy. Direct visualization of the fossa ovalis also supports selection of a puncture site appropriate for subsequent left atrial catheter manipulation.
- Cardiac function: baseline left- and right-ventricular function, relevant valvular disease, and the presence or absence of pericardial effusion should be documented.

### Step 2: guide transseptal puncture and PV cannulation

Optimal transseptal puncture targets the fossa ovalis. On TOE, the septum is assessed in the bicaval (90–110°) and short-axis (30–60°) views; biplane or three-dimensional imaging can further improve spatial orientation. Fluoroscopic guidance remains integral to advancement of the guidewire and transseptal system into the superior vena cava, while TOE provides complementary visualization of their relationship with the IAS. The needle and sheath are then withdrawn under continuous TOE visualization until tenting of the fossa ovalis is obtained. For this workflow, a mid-high-anterior puncture is generally sought to facilitate subsequent catheter manipulation. Orthogonal TOE views verify the intended superior-inferior and anterior-posterior position before puncture. Loss of tenting after needle advancement supports entry into the left atrium. After sheath advancement and de-airing, the guidewire is directed into the left upper PV using combined fluoroscopic and TOE guidance.

### Step 3: guide catheter configuration and positioning

Once transseptal access is obtained, the ablation catheter is introduced into the left atrium through the steerable sheath. Fluoroscopy and angiography remain central to guidewire navigation, PV cannulation, and confirmation of the conventional procedural landmarks. TOE is used as a complementary modality to track the catheter relative to the IAS, posterior wall, LAA, and target PV ostium. Each PV is cannulated by advancing the guidewire into the target vein under combined X-ray and TOE guidance, followed by advancement of the PFA catheter to the ostium. The specific contribution of TOE is real-time assessment of catheter configuration and its relationship with the ostium and adjacent soft-tissue structures, helping to identify deep advancement or unintended contact.

- **LUPV** is often cannulated first, requiring posterior-lateral sheath rotation.
- **LLPV** is assessed with slight caudal sheath angulation.
- **Right-sided PVs** require anterior rotation and rightward deflection, often visualized in bicaval or short-axis views.

Energy delivery follows the device-specific procedural protocol and is not determined by TOE alone. During each application, TOE can complement fluoroscopic/angiographic assessment by confirming that the catheter remains in the intended flower or basket configuration and by directly visualizing its apposition to the target PV ostium, without obvious spline overlap or deep entry into the vein. TOE may also facilitate confirmation that the device guidewire is positioned within the main branch of the intended target PV, rather than inadvertently entering a secondary branch of a previously treated vein. When posterior-wall ablation is performed, TOE can also document the catheter–wall relationship in the flower configuration.

### Step 4: post-ablation assessment

At the end of ablation, TOE provides an immediate anatomical safety assessment. The pericardial space is reassessed for new effusion, and the IAS is examined in mid-oesophageal short-axis and bicaval views with Colour Doppler to characterize the iatrogenic septal defect. If haemodynamic or electrocardiographic changes raise concern for coronary spasm or myocardial ischaemia, TOE can provide rapid assessment of global and regional ventricular function.^8^

## Practical considerations and future perspectives

This step-by-step approach provides cardiovascular imagers with a reproducible framework for integrating TOE into PFA and interacting with the electrophysiology team. Unlike TOE, ICE is established during AF ablation.^4,5^ Selection should reflect expertise, anaesthetic strategy, resources, and patient characteristics. ICE permits imaging under conscious sedation but requires a dedicated intracardiac catheter and additional disposable costs.^5^ TOE often entails deeper sedation or general anaesthesia but avoids an additional intracardiac imaging catheter. TOE applicability may be limited by oesophageal contraindications, including stricture, varices, active oesophagitis, or recent gastrointestinal bleeding.^7,9^ TOE and ICE should therefore be viewed as complementary strategies. TOE–fluoroscopy fusion may improve spatial orientation and device guidance; although useful in structural interventions, its role during PFA remains unvalidated.^10^

## Conclusions

A standardized TOE workflow provides complementary real-time soft-tissue information throughout fluoroscopy- and angiography-guided PFA, from pre-procedural assessment and transseptal access to catheter positioning and immediate safety evaluation. TOE should not replace conventional X-ray guidance but may enhance anatomical understanding. Evidence remains insufficient to establish superiority over ICE or reductions in radiation exposure, procedural time, complications, or cost. Prospective comparative studies are needed to define the incremental clinical value of TOE-guided PFA.

## Data Availability

No new data were generated or analysed in support of this research.

## Lead author biography

Federico Marchini, MD, is a cardiologist with expertise in multimodality cardiovascular imaging. His main clinical and research interests include valvular heart disease, infective endocarditis, and cardiomyopathies. He is actively involved in intraprocedural echocardiographic imaging, providing imaging guidance for both interventional cardiology and electrophysiology procedures. His clinical and research activity focuses on the integration of advanced cardiovascular imaging into procedural planning and intraprocedural guidance, with particular interest in the interaction between cardiovascular imaging and interventional and electrophysiological techniques.
